# An Unusual Zoonosis: Liver Abscess Secondary to Asymptomatic Colonic Foreign Body

**DOI:** 10.1155/2010/794271

**Published:** 2010-11-11

**Authors:** Justin S. Gundara, Richard Harrison

**Affiliations:** Department of Surgery, Wagga Wagga Base Hospital, University of New South Wales, NSW, Australia

## Abstract

A liver abscess may arise following any insult to gut integrity allowing portal drainage of bacteria to hepatocytes. Foreign bodies such as bones, toothpicks and items of stationery have previously been implicated in compromising gut epithelium. Here we present the case of a 57 year old man suffering from a left liver abscess. This was defined on CT which incidentally also identified a chicken bone protruding through the wall of the distal sigmoid colon. Whilst unwell with upper abdominal pain and sepsis, the presumed source of portal sepsis within the colon remained asymptomatic throughout. Following percutaneous drainage, the liver abscess resolved but the chicken bone had not passed at two months, necessitating atraumatic removal at colonoscopy. A high rate of incidental diagnoses suggests that unidentified foreign bodies may be vastly under recognised in cases of hepatic sepsis. Thus, identification of the precise mechanism of the liver insult demands thorough consideration; foreign body should be considered in all cases.

A liver abscess may arise following any insult to gut integrity that allows portal drainage of large bacterial showers to susceptible hepatocytes. Foreign bodies such as bones, toothpicks, and items of stationery have been implicated in compromising gut epithelium and may be encountered through unknowing ingestion or even psychiatric pica [1–7]. 

This case involved a 57-year-old Caucasian male presenting with a five-day history of progressive epigastric abdominal pain. This was punctuated by 48 hours of nausea, vomiting, and fever. Past medical history was significant only for an electively repaired left inguinal hernia. He had not undergone any recent travel, exposure to sick contacts or animals, and there was no history of trauma.

Upon examination, the patient appeared unwell and diaphoretic, with a fever of 38.7°C. Abdominal examination revealed generalised tenderness and focal epigastric peritonism. Blood tests were significant for a white cell count of 12.9 × 10^9^ (neutrophils: 11.1) and deranged liver function tests (bilirubin: 30; GGT: 311; ALP: 418; AST: 45; ALT: 58 units resp.). 

Computed tomography (CT) of the abdomen and pelvis showed a 7.3 × 5.8 cm irregular mass within liver segment III. The lesion possessed ill-defined margins and a partially cystic character, appearances consistent with liver abscess (Figure 1). 

Incidentally, within the pelvis, a linear density was also seen to be traversing the lumen of the mid sigmoid colon and extending into the presacral soft tissues at the S1 level. This object possessed the density of bony tissue (Figure 2).

On further questioning, the patient denied any unusual eating habits and could not recall recent ingestion of chicken or fish. He had not suffered any change in bowel habit, lower abdominal pain, or bleeding per rectum.

Following cultures, the patient was placed upon intravenous antibiotics and percutaneous hepatic drainage was undertaken. Abscess fluid failed to culture organisms, and the patient made a steady recovery with ongoing antibiotic therapy. The decision was made to observe the radio-opaque object in the hope that it would pass with bowel motions. 

Repeat CT at two months showed resolution of the liver abscess; the foreign body, however, had not passed. Colonoscopy was undertaken and identified what appeared to be an animal bone protruding through the wall of the colon in the region of the distal sigmoid, just above the rectosigmoid junction. A snare facilitated atraumatic removal. The patient was observed for 24 hours post procedure and discharged home without complication thereafter. He remained well at six-month follow-up. Further inspection of the foreign body revealed it to be a chicken bone (Figure 3).

Foreign body gut perforation and associated liver abscess is an increasingly recognised phenomenon [1, 2, 6]. This relationship is evolving such that some investigators now suggest that this “rare condition should be kept in mind when dealing with cases of hepatic abscess or even septic shock of unknown origin” [5]. 

Investigation of such cases has also been debated. This is of particular significance for patients possessing a paucity of risk factors for portal or systemic sepsis and those who remain asymptomatic from clinically quiescent, but obviously important foci of sepsis. Case reports of liver abscess with asymptomatic colonic foreign body and without recognition of ingestion have been previously reported twice [4, 6]. Authors stress that in such cases, CT is an invaluable diagnostic aid as a means of not only quantifying the liver insult but also identifying the foreign body of interest. Additionally, colonoscopy has been promoted as a useful diagnostic tool when investigating abscess aetiology [6]. Given that many foreign bodies will lodge in the foregut [2, 3, 8], perhaps this recommendation could be expanded to include the upper gastrointestinal tract.

Liver abscess and associated sepsis can be a serious and potentially life-threatening condition. Lacking previous reports and a high rate of incidental diagnoses (as demonstrated here) suggest that unidentified foreign bodies may be vastly underrecognised in cases of hepatic sepsis. Thus, identification of the precise mechanism of the liver insult demands thorough consideration and investigation. Foreign body should be considered in all cases. 

## Figures and Tables

**Figure 1:**
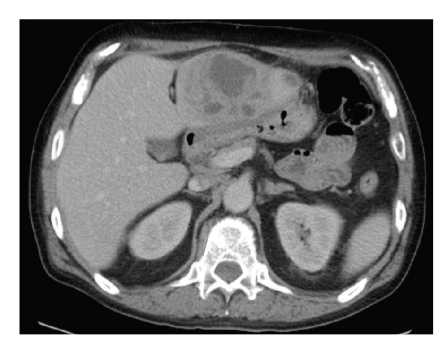
CT demonstrating left liver abscess.

**Figure 2:**
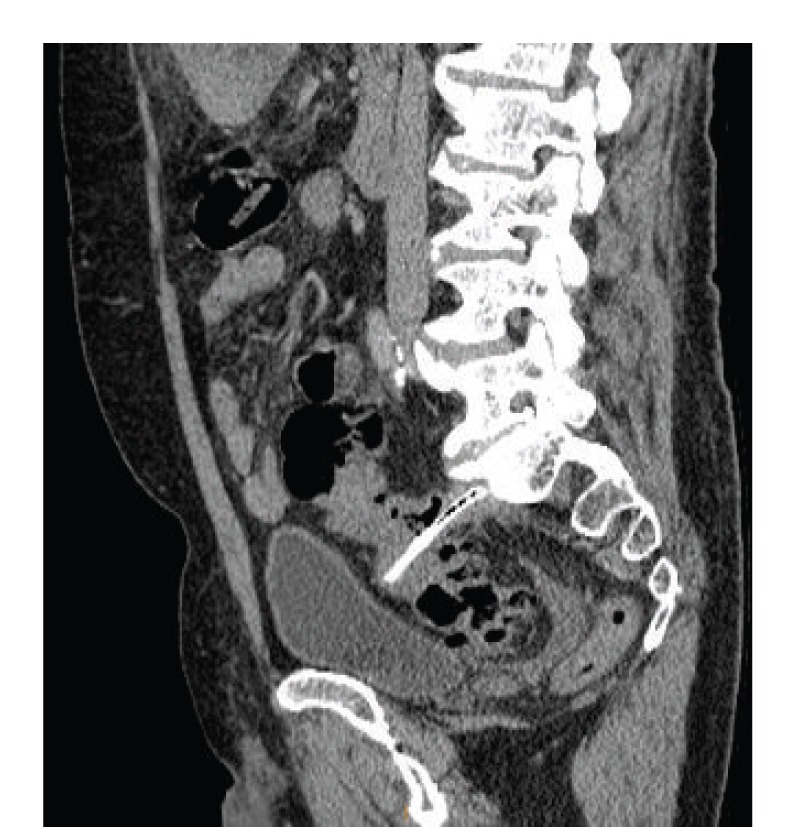
Sigmoid foreign body identified on CT.

**Figure 3:**
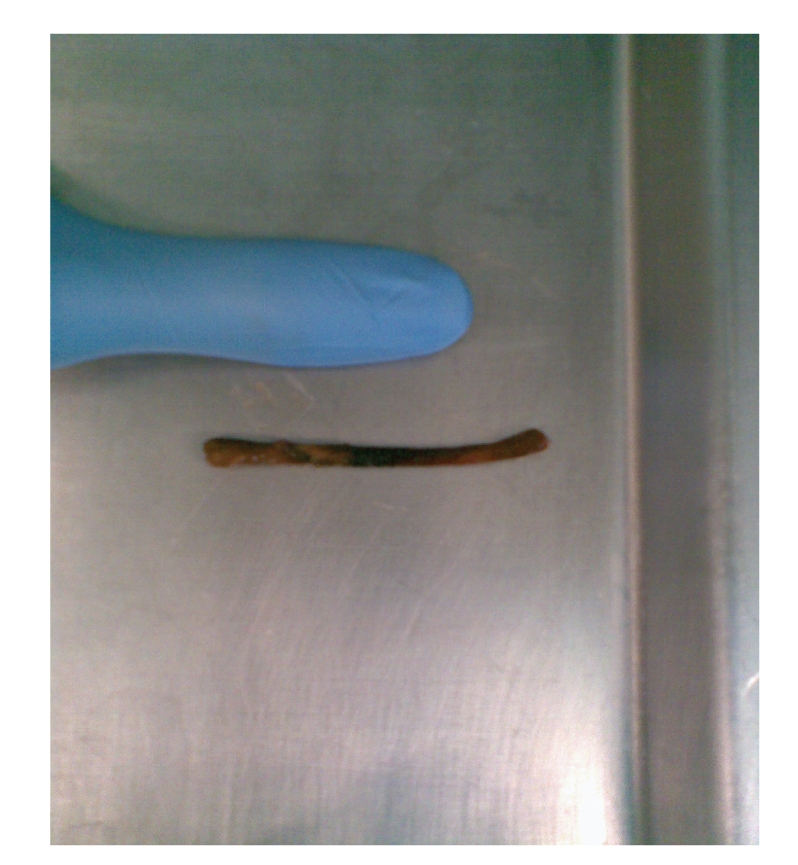
Chicken bone.
